# In Vitro Derived Dendritic Cells *trans*-Infect CD4 T Cells Primarily with Surface-Bound HIV-1 Virions

**DOI:** 10.1371/journal.ppat.0030004

**Published:** 2007-01-19

**Authors:** Marielle Cavrois, Jason Neidleman, Jason F Kreisberg, Warner C Greene

**Affiliations:** 1 Gladstone Institute of Virology and Immunology, University of California San Francisco, San Francisco, California, United States of America; 2 Department of Medicine, University of California San Francisco, San Francisco, California, United States of America; 3 Department of Microbiology and Immunology, University of California San Francisco, San Francisco, California, United States of America; King's College London, United Kingdom

## Abstract

In the prevailing model of HIV-1 *trans*-infection, dendritic cells (DCs) capture and internalize intact virions and transfer these virions to interacting T cells at the virological synapse. Here, we show that HIV-1 virions transmitted *in trans* from in vitro derived DCs to T cells principally originate from the surface of DCs. Selective neutralization of surface-bound virions abrogated *trans*-infection by monocyte-derived DCs and CD34-derived Langerhans cells. Under conditions mimicking antigen recognition by the interacting T cells, most transferred virions still derived from the cell surface, although a few were transferred from an internal compartment. Our findings suggest that attachment inhibitors could neutralize *trans*-infection of T cells by DCs in vivo.

## Author Summary

Dendritic cells (DCs) patrol peripheral mucosal sites, capturing and processing potential pathogens into antigenic peptides for presentation to T cells of lymphoid organs, and thereby initiating an immune response. HIV-1 had been proposed to use DCs as “Trojan horses,” hiding inside the DCs and surviving the degradation pathway to gain access to the lymph nodes and spread to the T cells. Our study challenges this “Trojan horse” model by showing that only HIV-1 virions bound to the surface of DCs, and not internalized virions, are transmitted to T cells. Even when T cells specifically recognized the antigen presented by DCs, the infection of T cells was principally mediated by virions remaining at the surface of the DCs. Interestingly, in this context of antigen-specific recognition, which increases the trafficking toward the immunological synapse of DC internal vesicles, where HIV-1 virions seem to hide, a few internal virions could infect T cells. Our findings suggest that in vivo transmission to T cells of HIV-1 virions captured by DCs should be more sensitive to neutralization than previously expected.

## Introduction

To ensure their survival, microbial pathogens have evolved strategies to subvert the action of cellular components of the host immune system, including dendritic cells (DCs). DCs patrol peripheral mucosal sites, capturing and processing potential pathogens into antigenic peptides for presentation by major histocompatibility complex (MHC) class II to CD4 T cells in lymphoid organs, initiating an immune response (for a review, see [1]). HIV-1 has been proposed to usurp this natural function of DCs to spread efficiently. HIV-1 entering the body via the mucosa and other peripheral sites may be transported by DCs to CD4 T cells deeper in the mucosa or in lymphoid organs [2–4]. HIV-1 that reaches lymphoid organs can also take advantage of the formation of DC–T-cell conjugates to promote its replication and spread [5–7].

DCs can transmit HIV-1 to T cells via two pathways. In the de novo pathway, DCs are actively infected with HIV, leading to the budding and spread of new virions to neighboring CD4 T cells. In the prevailing model of the *trans* pathway, intact HIV-1 virions are captured by alternative HIV-1 receptors, which bind virions without triggering fusion, and internalized into clustered compartments resembling late endosome/multivesicular bodies (MVBs) [8,9]. After interacting with a CD4 T cell, HIV-1–loaded DCs redistribute the virion-containing vesicles to the virological synapse [8,10,11]; CD4, CXCR4, and CCR5 receptors on T cells are recruited to this region, facilitating *trans*-infection [10].

How HIV-1 virions survive the uptake pathway designed to capture and cleave pathogens into peptides for antigen presentation remains unknown. HIV-1 could divert the intracellular trafficking of immunological synapse components to avoid degradation and thus survive until later transmitted to T cells. Alternatively, external virions could be transmitted to CD4 T cells since some HIV-1 virions remain deeply tangled in membrane protrusions and microvilli of the plasma membrane [8]. Using functional assays that detect virion fusion and productive infection of CD4 T cells, we investigated whether *trans*-infection is mediated through internalized or external HIV-1 virions in monocyte-derived DCs (MDDCs) and CD34-derived Langerhans cells (LCs).

## Results/Discussion

### Mature MDDCs Transmit HIV-1 to T Cells Primarily via the *trans* Pathway while Immature MDDCs Preferentially Transmit R5-Tropic HIV-1 via the De Novo Pathway

**Figure 1:**
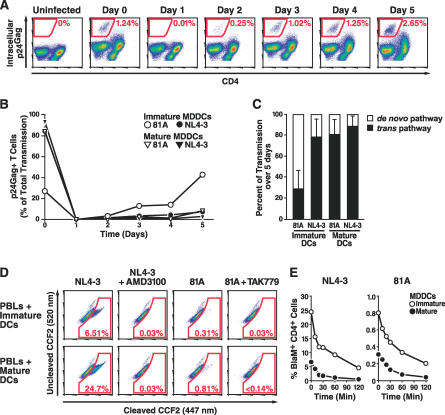
Mature MDDCs Transmit HIV-1 to T Cells Primarily via the *trans* Pathway (A–C) NL4–3 or 81A virions were bound to MDDCs. After washing, the cells were added to activated autologous T cells immediately or after 1 to 5 d of culture at 37 °C to allow HIV-1 transmission to T cells for 24 h. The number of transmission events was measured by monitoring the appearance of infected T cells detected by p24^Gag^ intracellular immunostaining. (A) Transmission of 81A virions from immature MDDCs to T cells over time. FACS plots represent the population of T cells (CD3^+^CD1a^–^) analyzed for intracellular Gag and CD4 expression. (B) Effects of maturation on HIV-1 transmission from MDDCs to T cells. Values are percentages of all transmission events over 5 d. (C) Relative contribution of *trans* pathway or de novo pathway in transmission of virus from immature or mature MDDCs to T cells. Data are averaged from MDDCs derived from nine donors. Transmission events from days 1 to 5 were added to determine the number of transmission events occurring via the de novo pathway. (D and E) NL4–3 or 81A virions containing BlaM-Vpr were bound to MDDCs at 4 °C. After washing, the cells were added to autologous T cells immediately (T_0_) or after incubation for 10 to 120 min at 37 °C. HIV-1 transmission was measured with a virion-based fusion assay after gating on CD3^+^CD4^+^ cells. (D) HIV-1 transmission from MDDCs to T cells at T_0_. FACS plots show CD3^+^CD4^+^CD1a^–^ cells analyzed for virion fusion. To control for specificity, MDDCs were incubated with T cells and entry inhibitors (500 nM TAK-779 or 500 nM AMD3100). (E) Effect of time on NL4–3 and 81A transmission from MDDCs to T cells. The curve is representative of four experiments.

The potential effects of the state of DC maturation and coreceptor utilization by HIV virions in the *trans* and the de novo pathways in HIV-1 transmission from DCs to T cells were evaluated. These studies were performed with immature MDDCs or MDDCs matured with tumor necrosis factor α and poly(I:C) [12] and with two laboratory-adapted viral strains, CXCR4-tropic NL4–3 and CCR5-tropic 81A (Figure 1). MDDCs were incubated with virions at 4 °C to promote viral binding and were then either added to autologous activated T cells immediately or incubated for 1 to 5 d at 37 °C before mixing the autologous T cells. The MDDCs were then incubated with T cells for 24 h to allow virion transfer to T cells. After an additional 2 d of incubation in the presence of azidothymidine (AZT), productive infection of T cells was measured by immunostaining with anti-p24^Gag^ (Figure 1A). Transmission of 81A (R5-tropic) virions from immature MDDCs to T cells was biphasic, as reported [11]. The early phase (0 to 1 d) involved the *trans* pathway; the later phase (1 to 5 d) involved the de novo pathway and was sensitive to the HIV protease inhibitor amprenavir (not shown). During the first day, 25% of R5-tropic 81A virions were transmitted by the *trans* pathway; 75% were transmitted by the de novo pathway over the ensuing 4 d (Figure 1B). In mature MDDCs, however, approximately 93% of virions were transmitted by the *trans* pathway during the first day. X4-tropic NL4–3 virions were transmitted by both immature and mature MDDCs principally by the *trans* pathway. Similar results were obtained when MDDCs were analyzed from nine different normal donors (Figure 1C).

In vivo, DCs may not immediately interact with T cells after virion capture. Accordingly, we investigated how a delay in T-cell contact might affect transmission through the *trans* pathway with a virion-based HIV-1 fusion assay [13,14]. MDDCs loaded with HIV-1 virions containing β-lactamase-Vpr (BlaM-Vpr) were incubated with autologous T cells, and fusion to CD4 T cells was monitored by the changes in fluorescence of CCF2, a BlaM substrate loaded into the cells (Figure 1D). When NL4–3 virions were presented immediately after binding to mature MDDCs, up to 24% of CD4 T cells displayed BlaM activity, indicating virion fusion. Transmission was less efficient when virions were presented by immature MDDCs. Fewer 81A than NL4–3 virions were transmitted, likely because there were fewer CCR5- than CXCR4-expressing cells in resting peripheral blood lymphocytes (PBLs). When virions were presented by MDDCs after incubation at 37 °C for up to 120 min, transmission efficiency decreased sharply (Figure 1E) in both immature and mature MDDCs. This rapid decrease was not due to a relative lack of sensitivity of the fusion assay in the context of *trans*-infection. As in our previous studies of T-cell infection with free virions, the fusion assay proved to be both sensitive and quantitative over a broad range of viral inputs in these DC–T-cell mixing experiments [13] (Figure S1). Thus, HIV-1 *trans*-infection from MDDCs to autologous CD4 T cells is efficient only for a limited time after virion capture.

Our results show that immature DCs preferentially transmit R5-tropic HIV-1 by the de novo pathway, as described [11,15–17], and X4-tropic HIV-1 by the *trans* pathway. Mature DCs transmit both R5- and X4-tropic virions mainly by the *trans* pathway. Since immature DCs are present at mucosal sites of viral entry, while mature DCs reside in lymph nodes and in the gut-associated lymphoid tissue (GALT), HIV-1 may exploit different transmission strategies at different anatomic sites in vivo. In healthy mucosa, immature DCs are likely to transmit R5-tropic HIV-1 principally via the de novo pathway, especially since the efficiency of the *trans* pathway declines rapidly (Figure 1E and [11,16]); the *trans* pathway might contribute to the local spread of virus from mucosal immature DCs to macrophages and CD4 T cells. However, in inflamed mucosal epithelium, which contains a greater proportion of mature DCs, HIV-1 transmission might preferentially involve the *trans* pathway, as in human cervical explants [4]. In lymph nodes and GALT, the proximity of mature DCs to T cells would further favor the *trans* pathway. Since DC–T-cell conjugates are major sites of HIV-1 production [5–7], *trans-*infection could be critical in the intense viral replication that characterizes the acute and chronic phases of untreated HIV-1 infection.

### Neutralizing Surface-Bound Virions on DCs Abrogates *trans*-Infection

**Figure 2:**
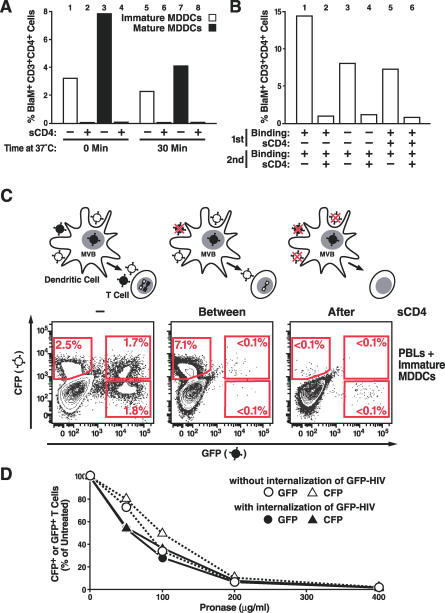
HIV-1 Virions Transmitted *In Trans* from MDDCs to T Cells Are Sensitive to sCD4 and Pronase (A) Immature and mature MDDCs were incubated at 4 °C with NL4–3 virions containing BlaM-Vpr, washed, and incubated for 30 min at 37 °C to allow virion internalization or held at 4 °C. MDDCs were treated (or not) with sCD4 at 4 °C to inactivate cell-surface virions. After extensive washes, loaded MDDCs were added to autologous PBLs to allow HIV-1 *trans*-infection of T cells. Fusion was measured with the virion-based fusion assay after gating on the CD3^+^CD4^+^ cells. The histogram presents a representative experiment independently performed five times with similar results. (B) sCD4 treatment of MDDCs loaded with HIV-1 virions does not inhibit transfer of virions loaded after treatment. Two sets of HIV-1 virions containing BlaM-Vpr were successively bound to immature MDDCs and neutralized with sCD4. After incubation with autologous CD4 T cells, fusion to CD3^+^CD4^+^ cells was assessed. (C) MDDCs were sequentially incubated with two reporter viruses (1 μg of p24^Gag^ each). GFP-HIV was bound and internalized, but CFP-HIV was bound only. Loaded MDDCs were incubated with autologous PBLs for 2 d. FACS plots show T cells analyzed for GFP and CFP expression when MDDCs were untreated or treated with sCD4 at 4 °C between or after loading of the reporter viruses. (D) GFP-HIV and CFP-HIV were either successively bound to MDDCs or GFP-HIV was, in addition, internalized by MDDCs during a 30-min incubation at 37 °C. MDDCs were then treated with increasing concentrations of pronase. Curves represent the number of T cells expressing CFP or GFP expressed as a percentage of the untreated samples. This graph presents a representative experiment with mature MDDCs that was independently performed three times with both immature and mature MDDCs.

To identify the cellular compartment from which HIV-1 is transmitted *in trans,* we selectively neutralized surface-bound HIV-1 virions with truncated recombinant soluble CD4 (sCD4; AIDS Reagent Program [18]), which binds the HIV-1 envelope gp120 protein and prevents engagement of CD4 on T cells. Cells were treated at 4 °C to protect internalized virions from sCD4 exposure. NL4–3 virions containing BlaM-Vpr were bound to MDDCs and allowed to internalize, and cell-surface virions were neutralized with sCD4. In the absence of an internalization step, surface-bound virions on immature and mature MDDCs effectively fused to CD3^+^CD4^+^ cells (Figure 2A; bars 1 and 3); this fusion was effectively blocked by sCD4 (bars 2 and 4). However, when HIV-1 virions were internalized at 37 °C for 30 min before treatment (bars 5 to 8), sCD4 still inhibited virion fusion (bars 6 and 8). To confirm that sCD4 neutralized surface-bound virions without impairing subsequent virion transfer, two sets of HIV-1 virions were successively bound to immature MDDCs, but only the first was neutralized with sCD4 (Figure 2B). Similar amounts of HIV-1 fused to T cells regardless of the presence of previously neutralized cell-surface virions on the DCs (bars 3 and 5). Thus, sCD4 does not interfere with virological synapse formation or the transfer of virions bound after sCD4 treatment.

To further confirm the absence of virion transfer from internal cellular compartments, we performed sequential loading of HIV-1 expressing green fluorescent protein (GFP) (GFP-HIV) and then HIV-1 expressing cyan fluorescent protein (CFP) (CFP-HIV) [19] (Figure 2C). GFP-HIV was bound to MDDCs and incubated at 37 °C to allow virion internalization. Some residual GFP-HIV virions remained at the surface. Next, CFP-HIV was bound but kept at 4 °C to prevent virion internalization. MDDCs were then incubated with autologous T cells for 48 h. sCD4 treatment after binding and internalization of GFP-HIV but before CFP-HIV binding fully blocked transmission of GFP-HIV (Figure 2C, middle panel), indicating that residual surface-bound GFP-HIV was the source of virus for transmission to T cells (left panel). Under these conditions, CFP-HIV was still transferred to T cells, confirming that sCD4 treatment did not affect subsequent transfer of virions and was not inherently harmful. In the absence of treatment, many double-positive GFP^+^CFP^+^ T cells were observed, indicating that more than one virion can be transmitted to each T cell (left panel).

Thus, the neutralization studies showed that only surface-bound, but not internalized, HIV virions mediated *trans*-infection from MDDCs to T cells. sCD4 likely neutralizes HIV-1 virions by competing for viral binding to the CD4 receptor on T cells, as intact HIV-1 virions, including gp120, remained associated with the MDDCs after treatment (unpublished data). Although sCD4 has been reported to allow HIV-1 fusion to cells that do not express CD4 [20], sCD4 did not induce fusion to CD4^–^ cells in our experiments, as demonstrated by the absence of BlaM transfer to CD8 T cells or B cells (not shown).

Next, we stripped surface HIV virions from MDDCs by proteolytic digestion (Figure 2D). GFP-HIV was again allowed to bind and internalize. CFP-HIV was restricted to the surface of MDDCs and served as a control for neutralization by the proteolytic enzymes. MDDCs loaded with GFP-HIV and CFP-HIV were then treated with trypsin as described [10,21] or pronase and incubated for 2 d with T cells. Trypsin treatment did not effectively remove externally bound CFP-HIV virions (unpublished data). However, in the presence of increasing amounts of pronase, fewer surface-bound CFP-HIV virions were transmitted to T cells, indicating increasingly effective removal the surface-bound HIV-1 by this protease cocktail. However, pronase had the same effect on the transfer of GFP-HIV, whether it had been internalized or not. Thus, successfully transmitted GFP-HIV virions appear to originate from the cell surface rather than from an internal, pronase-resistant compartment in MDDCs.

We considered the possibility that the low levels of transmission in the presence of sCD4 could correspond to transmission events from internalized HIV-1, masked under our experimental conditions. However, changes in viral input, internalization time, and viral strains failed to reveal significant transfer from internal compartments (Figure S2). We also studied a second type of DC, LCs derived from CD34^+^ cord blood cells (MatTek; http://www.mattek.com). As observed in MDDCs, the transfer of HIV virions from LCs to allogenic T cells again was mediated by virions bound at the surface of the LCs (Figure S3). Since MDDCs and LCs derived from CD34 progenitors are excellent surrogates of in vivo DCs, we conclude that most virions transmitted *in trans* in vivo likely originate from the cell surface.

These results in MDDCs and LCs sharply contrast with the report that formed the basis for the prevailing model of *trans*-infection [21]. In that report, surface-bound virions were neutralized by proteolytic digestion with trypsin. Although in our hands trypsin did not digest surface-bound virions as potently as pronase, the discordance of results likely lies in the use of different reporter systems. Kwon et al. [21] used a luciferase reporter that does not allow the distinction between infection of T cells or MDDCs. Since the immature MDDCs used in that study are highly susceptible to fusion with the R5-tropic BaL envelopes [12] and efficiently replicate CCR5-tropic HIV-1 [15,22], the luciferase activity may have derived from infected immature MDDCs, not T cells, in the coculture. Our flow-based assays, which permit a clear distinction between infection of T cells and MDDCs in coculture, reveal that HIV-1 virions transmitted *in trans* are sensitive to pronase treatment.

Our results also differ from three later studies, further supporting the notion that HIV infection of T cells by DCs involves the transfer of internalized virions from DCs to interacting CD4 T cells [9,10,15]. McDonald et al. [10] showed the recruitment of “trypsin-resistant” HIV-1 virions to the immunological synapse; however, functional assays were not performed to confirm that the interacting CD4 T cells were actually infected by the recruited virions. We suspect that, while virions may be transported to the synapse, these virions are not successfully transmitted. Wiley et al. [9] showed the release of infectious virions from HIV-1–loaded MDDCs, even after surface-bound virions were removed with trypsin. However, the efficacy of the trypsin treatment was only controlled in the experiments measuring the release of p24^Gag^ in the supernatant, not in studies measuring the infectivity of these virions. Finally, Ganesh et al. [15] observed the transfer of some virions from MDDCs to T cells in the presence of neutralizing antibodies, a surprising result in light of our findings with sCD4. In their study, the efficacy of the antibody neutralization was measured with free virions but not with MDDCs bearing only surface-bound HIV-1 virions. The amount of antibody required to neutralize free virions might be lower than the amount needed to neutralize surface-bound virions on MDDCs, a possibility that would explain our divergent results. Of note, our findings are supported by a recent study showing that, in Raji cells expressing DC-SIGN (DC-specific ICAM-3 grabbing nonintegrin), surface-bound rather than internalized virions are transmitted *in trans* to 293T cells expressing CD4 and CCR5 [17].

### *trans*-Infection in the Context of Superantigen Stimulation

**Figure 3:**
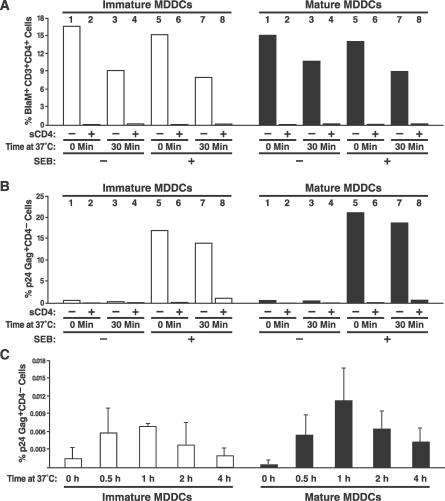
HIV-1 Transmitted from SEB-Stimulated MDDCs to T Cells Remains Mostly Sensitive to sCD4 NL4–3 virions containing BlaM-Vpr were bound to SEB-pulsed MDDCs. The MDDCs were then incubated or not at 37 °C for the indicated time, treated or not with sCD4 at 4 °C, and incubated with purified autologous CD4 T cells. (A–C) Viral transfer was measured by analyzing HIV-1 fusion to CD4^+^CD3^+^ T cells after 2 h of culture (A) and by measuring productive infection of the T cells by intracellular Gag immunostaining 3 d later (B and C). (B) Histogram depicts one representative experiment performed three times with cells from three independent donors. (C) HIV-1 transfer to resting T cells from DCs pulsed with SEB and treated with sCD4 over time. This experiment was performed in triplicate; similar results obtained with MDDCs from two additional donors.

Several reports have suggested that captured HIV-1 virions are stored in MVBs, raising the possibility that HIV-1 mediates *trans*-infection of T cells by highjacking a pathway involved in the trafficking of internal vesicles to the immunological synapse. In DCs, the transport of MHC class II from the MVB to the immunological synapse requires a T-cell–mediated signal [23]. Only T cells of the appropriate antigen specificity trigger this transport. Since antigen recognition could mobilize the release of HIV-1 virions from the MVB, we investigated *trans*-infection in the context of stimulation with a superantigen, staphylococcal enterotoxin B (SEB) (Figure 3). SEB activates T cells by crosslinking the variable region of T-cell receptor β-chain and the MHC class II molecule expressed on the DC surface [24].

NL4–3 virions containing BlaM-Vpr were bound at 4 °C to SEB-pulsed MDDCs. Viral transfer to autologous purified CD4 T cells was measured after allowing virion internalization, or not, by MDDCs. Again, sCD4 completely blocked HIV-1 transmission (Figure 3A). However, since the fusion assay does not require T-cell activation to generate a positive signal, *trans*-infection could be detected in the absence of engagement of the T-cell receptor and MHC class II. To further ensure that transfer was analyzed only when the MDDCs and T cells were effectively engaged, we measured productive infection of resting T cells by immunostaining for p24^Gag^ (Figure 3B). Only T cells stimulated by SEB-loaded MDDCs are rendered permissive by releasing a postentry restriction block created by APOBEC3G (apolipoprotein B mRNA-editing enzyme, catalytic polypeptide-like 3G) [25]. Again, the vast majority of transmission events were neutralized by sCD4. However, under these experimental conditions, a few virions were transmitted from an sCD4-resistant compartment, as evidenced by the slight increase in transfer when SEB-loaded MDDCs were allowed to internalize HIV-1 virions at 37 °C. These transmission events were not due to new virion production by MDDCs since similar results were observed when AZT was added after 24 h of coincubation of MDDCs and T cells. When SEB-pulsed MDDCs were allowed to internalize HIV-1 virions for a longer period, HIV-1 virion transfer slightly increased peaking at 1 h of internalization (Figure 3C). Subsequently, the efficiency of transfer from internal compartments decreased, likely as a consequence of degradation or inactivation of the internalized virions.

In conclusion, HIV-1 virions transmitted *in trans* from DCs to T cells principally originate from the surface of DCs, except during antigen recognition, when a few internalized virions may also be transmitted to the antigen-specific T cells. Whether these rare events contribute to the preferential infection and elimination of HIV-specific T cells in vivo [26] is not known. Nevertheless, even within this context, the vast majority of transmitted virions are derived from the surface of DCs. Our results do not challenge the prevailing view that HIV-1 virions are internalized in the DCs. Indeed, we detected large amounts of internalized HIV-1 virions by microscopy. However, unless HIV-1–loaded DCs encounter T cells of the appropriate specificity, virion internalization appears to be a dead end for HIV-1 *trans*-infection. Since the C-type lectin receptors involved in *trans*-infection are localized in lipid rafts [27], surface-bound HIV-1 likely exploits the clustering of lipid rafts at the immunological synapse to enhance *trans*-infection of CD4 T cells. Because *trans*-infection principally involves surface-bound virions, our findings suggest that attachment inhibitors could be used to limit *trans*-infection of T cells by DCs, in vivo.

## Materials and Methods

### Two-phase transmission of HIV-1 from MDDCs to autologous T cells.

To study HIV-1 transmission from DCs to T cells, MDDCs (2 × 10^6^) were incubated with 81A or NL4–3 virions (50 μg of p24^Gag^ /ml) for 1 h at 4 °C, washed four times in cold PBS, incubated for 0 to 5 d at 37 °C, diluted 1:10, and added to autologous phytohemagglutinin-activated PBLs (2 × 10^6^). Cocultures were maintained for 3 d in RPMI with 10% FBS, 20 IU/ml IL-2, 25 ng/ml IL-4, 50 ng/ml GM-CSF, and penicillin and streptomycin (100 μg/ml each); at 24 h, AZT (10 μM) was added to prevent further infection. Infected T cells were identified by intracellular immunostaining for p24^Gag^ combined with antibodies against CD3, CD4, and CD1a. Productively infected T cells represent the percentage of p24^Gag+^CD4^–^ cells in the CD3^+^CD1a^–^ population and correspond to infected CD4 T cells that had effectively downregulated CD4 receptors due to expression of select viral gene products, including *nef, vpu,* and *env* [28]. In some experiments, an HIV protease inhibitor, amprenavir (40 nM) (Division of Acquired Immunodeficiency Syndrome, National Institute of Allergy and Infectious Diseases; http://www.niaid.nih.gov), was added during the binding step and maintained for the rest of the experiment.

### Measuring *trans*-infection of autologous T cells by MDDCs.

MDDCs derived from CD14^+^ monocyte were induced to mature with poly(I:C) and tumor necrosis factor α [12]. The 81A or NL4–3 virions containing BlaM-Vpr (500 ng of p24^Gag^) [12–14] were incubated with MDDCs (2 × 10^6^) or with CD34-derived LCs for 1 h at 4 °C, washed four times in cold PBS, and incubated at 37 °C for the indicated time to allow virion internalization or kept at 4 °C. Aliquots (2 × 10^5^ cells) were added to autologous resting PBLs (2 × 10^6^), and incubated at 37 °C for 1 h. HIV-1 fusion to CD4^+^CD3^+^ cells was measured using the virion-based fusion assay combined with immunostaining with CD1a-APC, CD4-PE Cy7, and CD3-APC Cy7 antibodies [13,14]. Cells were analyzed by flow cytometry (BD LSRII; Becton Dickinson, http://www.bd.com) and analyzed with FlowJo software (Treestar Software, http://www.flowjo.com).

### Assessing *trans*-infection of autologous T cells by MDDCs by measuring proviral expression.

GFP-HIV virions were bound to MDDCs for 1 h at 4 °C and allowed to internalize at 37 °C for 30 min; CFP-HIV virions were only bound to MDDCs. As indicated, surface virions were neutralized with sCD4 before or after the binding of CFP-HIV or by pronase after the binding of GFP-HIV and CFP-HIV. MDDCs were then incubated with autologous T cells for 48 h. Cells were immunostained with CD1a-APC, CD4-PE Cy7, and CD3-APC Cy7 antibodies and analyzed by flow cytometry.

### Neutralization of surface-bound virions.

To neutralize surface-bound virions, MDDCs or CD34-derived LCs loaded with HIV-1 virions were incubated for 90 min at 4 °C with 20 μg/ml sCD4 in RPMI and 10% FBS and extensively washed with PBS before MDDCs or CD34-derived LCs were added to T cells. To neutralize virions with pronase, the HIV-1–loaded MDDCs were incubated for 30 min at 4 °C with 50 to 400 μg/ml pronase (Roche, http://www.roche.com).

### Measurement of *trans*-infection of autologous CD4 T cells by SEB-pulsed MDDCs.

MDDCs (2 × 10^6^) were pulsed with SEB (0.5 μg/ml) at 37 °C for 1 h. NL4–3 virions containing BlaM-Vpr (500 ng of p24^Gag^) were allowed to bind at 4 °C to the MDDCs; as indicated, cells were incubated for 30 min to 4 h at 37 °C to allow internalization. Surface-bound virions were then neutralized or not with sCD4. The HIV-loaded MDDCs were added to autologous purified resting CD4 T cells, and *trans*-infection was measured with the fusion assay at 2 h or by measuring productive infection after 3 d of coculture. Productively infected T cells were identified by intracellular immunostaining for p24^Gag^ combined with antibodies against CD3, CD4, and CD1a. We then measured the percentage of p24^Gag+^CD4^–^ cells in the CD3^+^CD1a^–^ population.

## Supporting Information

Figure S1 The Virion-Based Fusion Assay Is Sensitive and Quantitative in the Context of *trans*-Infection Serial dilutions of NL4–3 virions containing BlaM-Vpr were bound to immature or mature MDDCs at 4 °C. After three washes, these HIV-1–loaded MDDCs were incubated with autologous T cells for 2 h. Viral transfer to T cells was determined by measuring the amount of BlaM^+^ T cells.
(50 KB PDF)

Figure S2 Changes in Virion Input, Internalization Time, and Viral Strain Fail to Reveal HIV-1 *trans*-Infection of T Cells from Internal Compartments Serial dilutions (A) or 500 ng (B and C) of virions containing BlaM-Vpr was bound to MDDCs. Where indicated, viral strains other than NL4–3 were used (C). Virions were then internalized (or not) at 37 °C for 30 min (A and C) or for increasing amounts of time (B). After sCD4 treatment at 4 °C to inactivate surface virions, the loaded DCs were incubated with PBLs. Curves represent the transfer of HIV-1 from immature or mature MDDCs to T cells in the presence or absence of sCD4 treatment.
(68 KB PDF)

Figure S3 LCs Transmit HIV-1 to T Cells from an External Compartment (A) Phenotype of the CD34-derived LCs matured or not with 5 μg/ml LPS and 50 ng/ml tumor necrosis factor α for 24 h.
(B and C) NL4–3 virions containing BlaM-Vpr were bound to immature or mature LCs at 4 °C. After washing, the cells were added to allogenic T cells immediately or after incubation at 37 °C as indicated. HIV-1 transmission to T cells was measured with the virion-based fusion assay after gating on the CD3^+^CD4^+^ cells.
(B) Effect of time on HIV transfer from immature or mature LCs to T cells. Histogram shows one of two experiments.
(C) LCs were treated or not at 4 °C with sCD4 before incubation with the T cells.
(113 KB PDF)

## Abbreviations

AZT: azidothymidine
CFP: cyan fluorescent protein
DC: dendritic cell
GALT: gut-associated lymphoid tissue
GFP: green fluorescent protein
LC: Langerhans' cell
MDDC: monocyte-derived dendritic cell
MHC: major histocompatibility complex
MVB: multivesicular body
PBL: peripheral blood lymphocyte
sCD4: soluble CD4
SEB: staphylococcal enterotoxin B
