# Supplementary material for: In Vitro Derived Dendritic Cells trans-Infect CD4 T Cells Primarily with Surface-Bound HIV-1 Virions
Source: PLoS Pathog. 2007 Jan 19;3(1):e4. doi: 10.1371/journal.ppat.0030004 (PMC1779297; doi:10.1371/journal.ppat.0030004)
Supplement: Supplementary file 1 [file ppat.0030004.sg001.pdf]

Figure S1

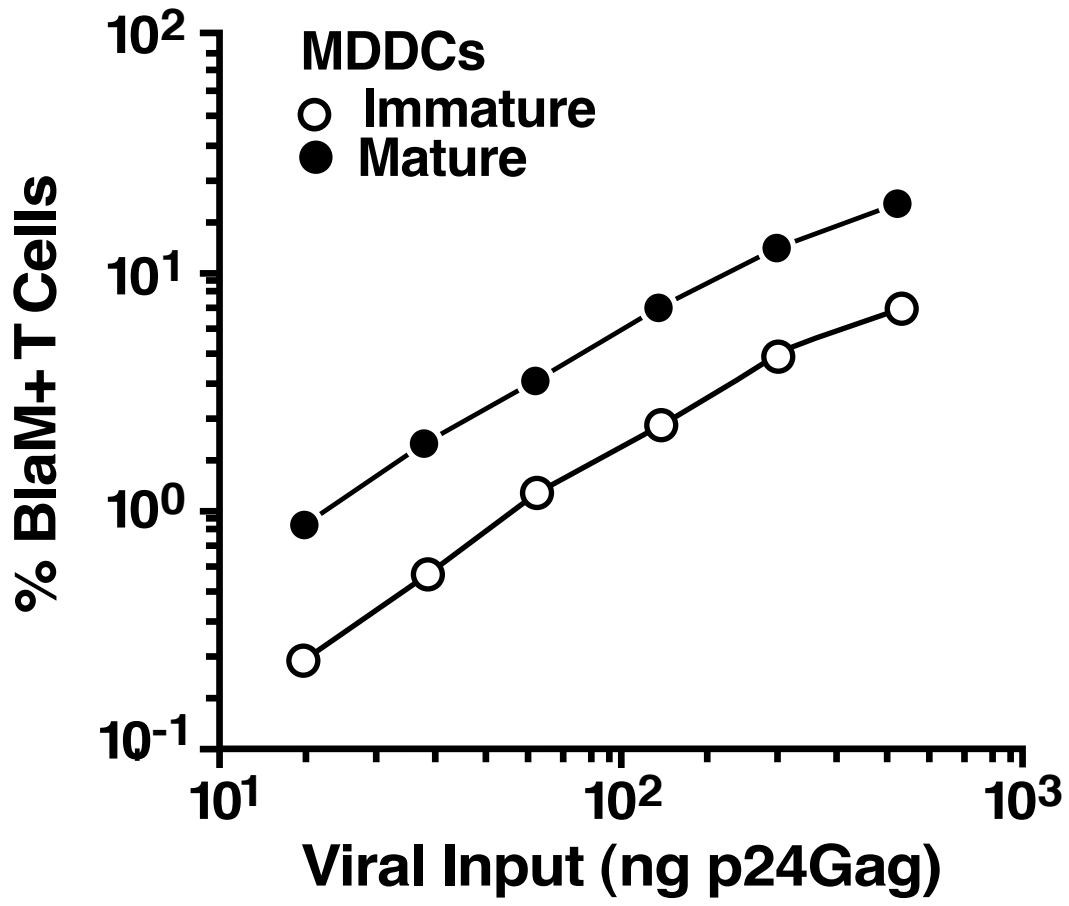
MDDCs
Immature
Mature
% BlaM+ T Cells
10^2
10^1
10^0
10^{-1}
10^1
10^2
10^3
Viral Input (ng p24Gag)
