# Supplementary material for: In Vitro Derived Dendritic Cells trans-Infect CD4 T Cells Primarily with Surface-Bound HIV-1 Virions
Source: PLoS Pathog. 2007 Jan 19;3(1):e4. doi: 10.1371/journal.ppat.0030004 (PMC1779297; doi:10.1371/journal.ppat.0030004)
Supplement: Supplementary file 2 [file ppat.0030004.sg002.pdf]

Figure S2

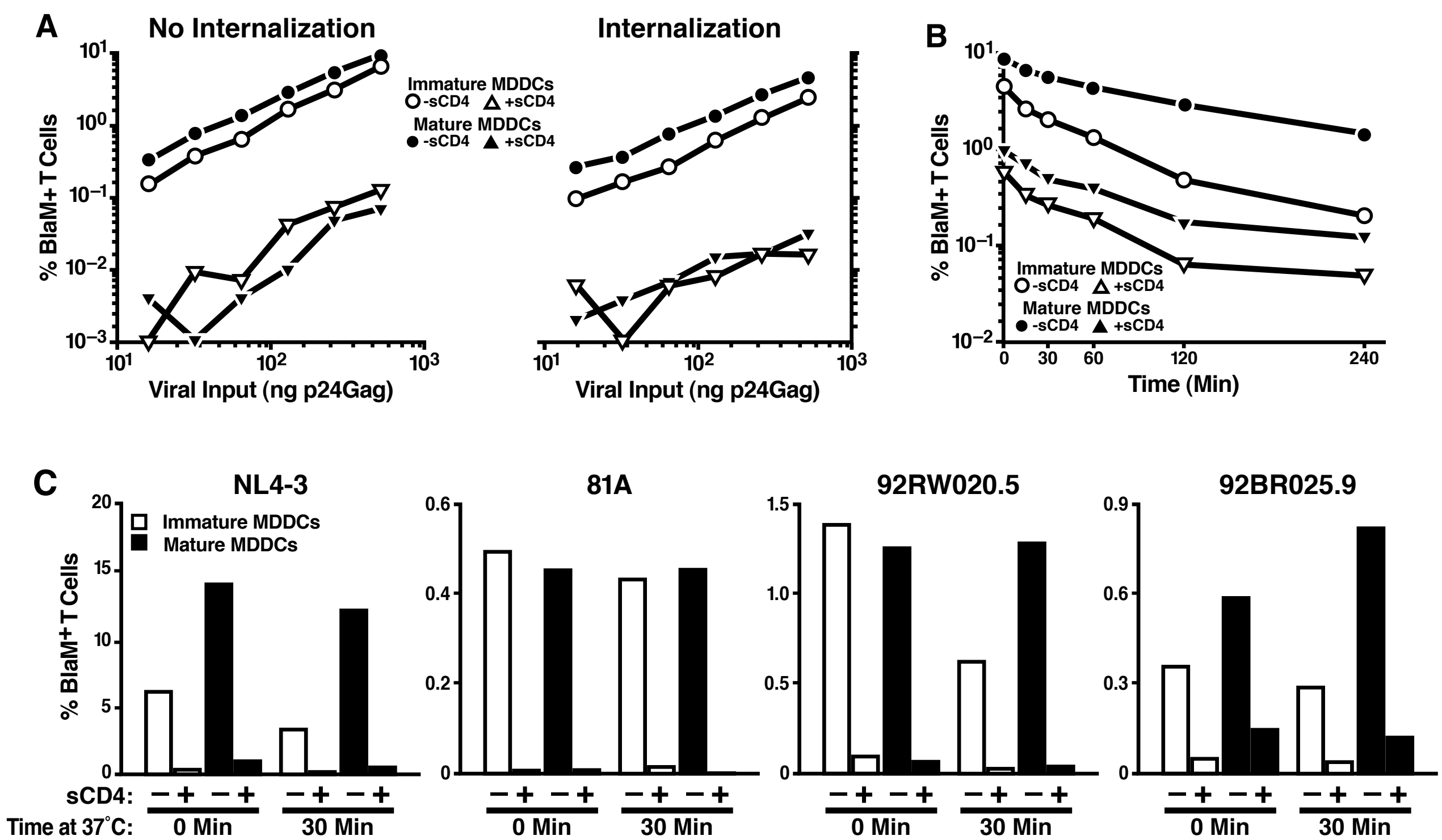

A
No Internalization
Internalization
% BlaM+ T Cells
Viral Input (ng p24Gag)
Immature MDDCs
-sCD4 +sCD4
Mature MDDCs
-sCD4 +sCD4
B
% BlaM+ T Cells
Time (Min)
Immature MDDCs
-sCD4 +sCD4
Mature MDDCs
-sCD4 +sCD4
C
NL4-3
81A
92RW020.5
92BR025.9
Immature MDDCs
Mature MDDCs
% BlaM+ T Cells
sCD4:
Time at 37˚C:
0 Min
30 Min
