# Supplementary material for: In Vitro Derived Dendritic Cells trans-Infect CD4 T Cells Primarily with Surface-Bound HIV-1 Virions
Source: PLoS Pathog. 2007 Jan 19;3(1):e4. doi: 10.1371/journal.ppat.0030004 (PMC1779297; doi:10.1371/journal.ppat.0030004)
Supplement: Supplementary file 3 [file ppat.0030004.sg003.pdf]

Figure S3

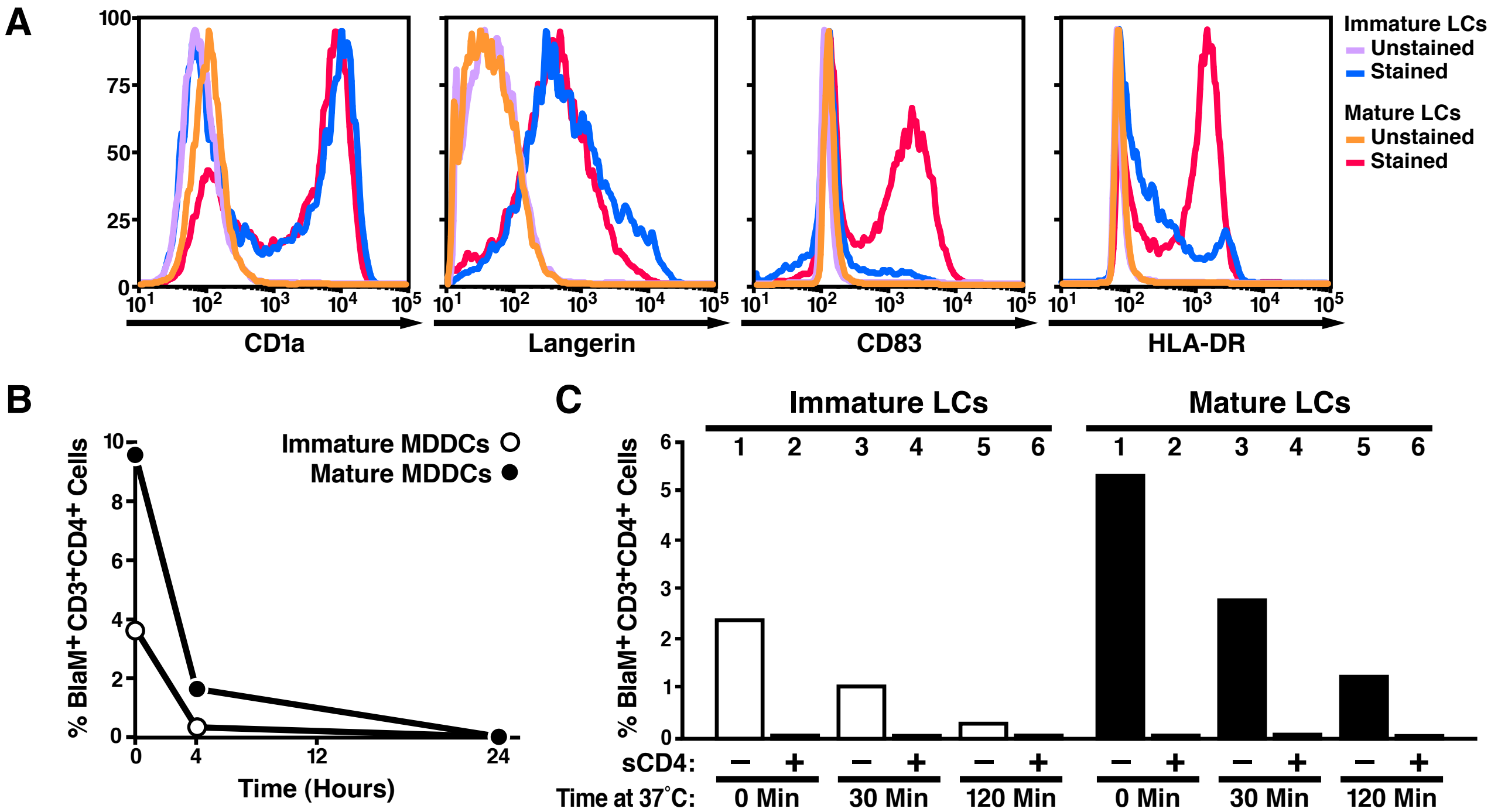
A
100
75
50
25
0
CD1a
Langerin
CD83
HLA-DR
Immature LCs
Unstained
Stained
Mature LCs
Unstained
Stained
B
% BlaM+ CD3+CD4+ Cells
Immature MDDCs
Mature MDDCs
Time (Hours)
0
4
12
24
C
Immature LCs
Mature LCs
1 2 3 4 5 6
sCD4:
Time at 37˚C:
0 Min
30 Min
120 Min
